# Promoting labour market inclusion of the chronically ill: a scoping review of Scandinavian countries’ efforts

**DOI:** 10.1177/14034948221096005

**Published:** 2022-05-10

**Authors:** Håvard Thorsen Rydland, Henrik Litleré Bentsen, Rune Ervik, Kjersti Grønning, Kamrul Islam, Egil Kjerstad, Tord Skogedal Lindén

**Affiliations:** 1 NORCE Norwegian Research Centre AS, Bergen, Norway; 2 Department of Public Health and Nursing, Faculty of Medicine and Health Sciences, Norwegian University of Science and Technology, Trondheim, Norway

**Keywords:** Work inclusion, chronic disease, Scandinavia, return to work, review

## Abstract

**Objectives:**

This article is a scoping review of efforts in labour market inclusion of the chronically ill in the Scandinavian countries, a research area that has received much political as well as research attention in recent years. The aim of the review was to identify promising strategies and the need for further research.

**Methods:**

Six electronic databases were searched for literature published between 2015 and 2020. We included peer-reviewed articles that studied the effect of measures, aimed at the workplace or at the individual, that are intended to increase participation. Our search resulted in 2718 articles; our screening procedures resulted in 47 included articles.

**Results:**

Among the included studies, musculoskeletal problems (17 articles) and mental health problems (29 articles) were the most frequent chronic conditions. Multimodal occupational rehabilitation programmes directed towards the individual employee were the most frequent interventions (30 articles). Return to work (24 articles) and sickness absence (12 articles) were the most common outcomes. About half (25 articles) of the included studies reported a positive impact of the intervention on work inclusion of the chronically ill.

**Conclusions:**

Our review found little evidence of how government programmes directed towards the supply side of the labour market succeed in including the chronically ill. Our review further indicated that multidisciplinary workplace interventions have a substantial effect. We also identified a significant lack of research on the effect of various governmental policies and programmes, including local health, work and welfare services, and limited coordination and cooperation between health and work services professions.

## Introduction

Scandinavian countries (Scandinavia refers to Norway, Sweden and Denmark while the term Nordic countries would include Finland and Iceland as well) are among the highest public spenders on sickness and disability benefits in the Organisation for Economic Co-operation and Development (OECD) at 3–5% of the gross domestic product. This could in part be explained by the countries’ benefit generosity and high coverage rates, but rates are nevertheless high, substantially surpassing spending on unemployment. Although unemployment in Scandinavia is comparatively low, the relative disadvantage of people with a disability is similar to the OECD average [1]. With future prospects of shrinking and ageing populations, increasing the labour supply – particularly among people with health challenges – is presented as a solution to achieve fiscal balance. Investments in work-related initiatives for improving health and labour market inclusion for these groups could contribute significantly to meet this challenge [2]. Moreover, Scandinavian countries rely heavily on high employment to raise revenues for their comprehensive welfare states [3].

People with chronic illness represent an important target for these initiatives; their employment rate is low, but with adequate adjustments, many could function at work. In this article, we follow the simple approach of Bernell and Howard [4] towards the chronic disease term: we do not restrict it to specific diagnoses, but rather diseases, conditions, and syndromes which are continuously present or reoccurring. The potential for successful implementation of work-promoting measures should be high in welfare states with a tradition for active labour market policies and obligations to an inclusive working life [5]. This scoping review therefore maps Scandinavian efforts in promoting labour market inclusion of the chronically ill. The aim of the review is to identify promising strategies and the need for further research. Norway, Sweden and Denmark have traditionally been fairly similar with regard to tax levels, welfare state spending and generosity, and labour market participation, although with some differences emerging over the past decades [6].

Comparative research suggests that features associated with Scandinavian welfare states, such as active labour market policies and high social spending, are beneficial for work inclusion of the chronically ill [7–9]. However, reducing under-employment of these groups is important to improve their income security, as well as contributing towards financial sustainability of the Scandinavian welfare model. In addition, as displayed in a recent review by Proper and van Oostrom [10], workplace interventions can improve the health and wellbeing of the affected employees.

Using self-reported data from the repeated cross-sectional Norwegian Survey of Living Conditions, Van der Wel et al. [11] demonstrate that health-related unemployment has been rising in Norway since the 1980s, and that economic recessions appear to strengthen this association. Results from Heggebø [12, 13] indicate that health selection to employment (i.e. that one’s health condition influences the probability of becoming and staying employed) is stronger in Denmark compared with Norway and Sweden, possibly as a consequence of the Danish ‘flexicurity’ model.

On the one hand, this health selection to employment may occur due to employers’ preference for healthy employees, as they may see health as a proxy for productivity [12]. On the other hand, this pattern may reflect how people with health challenges are unable to cope with the demands of working life and leave the labour force more or less (in)voluntarily. In either case, most modern welfare states have arrangements for promoting labour market participation among ill and disabled people. These arrangements and their effects are at the core of the present review study.

Return-to-work (RTW) is the internationally accepted term for all activities that enable and facilitate return to work after an illness. It includes people-oriented or workplace-oriented intervention programmes, rehabilitation programmes, and training tools (such as cognitive behavioural therapy, increasing physical activity, workplace adaption [14]. Facilitating RTW can be challenging due to the complexity of work disability, in which several factors can be expected to influence RTW independent of injury and illness. Examples of such factors include gender, age, education, socioeconomic status, self-efficacy and expectations for recovery and RTW, work demands, RTW coordination, multidisciplinary interventions [15], encounters between patients and health/RTW professionals [16–18] and roles, beliefs and perceptions of various stakeholders [19]. Being able to work has been emphasised as meaningful by chronically ill people, despite time periods out of the workplace [20].

Several earlier reviews have summarised and synthesised knowledge on the work inclusion of the chronically ill [14, 19, 21–31]. These earlier review studies provide important insights on successful strategies but vary as to whether they cover Norway, Sweden and Denmark. Our study, however, offers a review of how Scandinavian countries promote labour market participation of the chronically ill based on articles published in the last 5 years – from 2015 to 2020. By concentrating on this time period, we avoid possible negative impacts from the financial crisis of 2008 and its aftermath, hence covering a more ‘normalised’ labour market situation.

With a focus on three countries belonging to the same welfare regime, our study complements existing reviews with an up-to-date overview of how people with health challenges can be included in paid employment in generous and encompassing welfare states with comparatively low general unemployment. Our scoping review includes a wide range of intervention types, conditions and methodology, aiming to draw a rich map of work inclusion efforts in Scandinavia. The purpose of this review is to identify promising strategies and the need for further research within this area.

In the following, we first offer a brief review of how Norway, Sweden and Denmark promote labour market participation of people with reduced working capacities. Then we describe our methods and procedures in the searching and screening for articles. Our next step is to use relevant categories to describe the results, that is, the articles in the final text corpus. Finally, we discuss our findings’ relevance for research and practice.

## Promoting labour market participation of people with reduced working capacities in Norway, Sweden and Denmark

A governmentally appointed commission in Norway recently reported on measures to increase employment [3, 32]. As part of their work, they describe important characteristics of Nordic welfare states, and summarise research and trends across Nordic countries. These countries have recently implemented reforms to increase employment rates. In both Sweden and Denmark, eligibility criteria in several income security schemes have been tightened [32].

Despite many similarities between the countries there are some differences. While unemployment benefits are administered by the state in Norway, trade unions in Sweden and Denmark have their own systems. Active labour market policy (ALMP) is a local responsibility in Denmark whereas the state takes care of this in Norway and Sweden. Employment protection is more limited in Denmark than in Sweden and Norway. The Danish flexicurity model combines limited employment protection with generous income security schemes [3]. Moreover, comparing Norway and Sweden, Næsheim and Sundt [33] found that the higher employment rate for disabled people in Sweden compared with Norway, could partly be due to a higher state employment rate for people with disabilities in Sweden than in Norway. A higher share of people in Norway than in Denmark and Sweden draw on health-related benefits. When it comes to measures to promote labour market participation, the Norwegian governmentally appointed commission points to how Sweden, to a greater extent than Norway, draws on wage subsidies that compensate for reduced productivity to include groups with reduced working capacities. In 2019, 2.2% of the workforce in Sweden drew on wage subsidies compared with 0.3% in Norway where focus has been set more on competence building. Although the commission does not compare Norway with Denmark and Sweden in this respect, it states that individual adaptation for jobseekers is infrequently used in Norway. ALMP is applied in all Nordic welfare states, but according to the commission, Denmark and Sweden use this to a higher extent than Norway. Examples of such ALMPs promoted by the commission include close follow-up of people with reduced working capacities or combining medical treatment with work-related support for this group [3]. A Danish website systematising experiences and research on ALMPs is also emphasised as helpful in promoting labour market participation (jobbeffekter.dk). The compensation rate of Norwegian sickness benefit is more generous than in Sweden and Denmark, and employers in Sweden and Denmark also have stronger responsibility for financing this benefit than their Norwegian counterparts. Although such comparisons must be exercised with care, sickness absence in Norway is higher than in other countries, and the design of the sickness benefit scheme contributes to this.

## Methods

**Figure 1.**
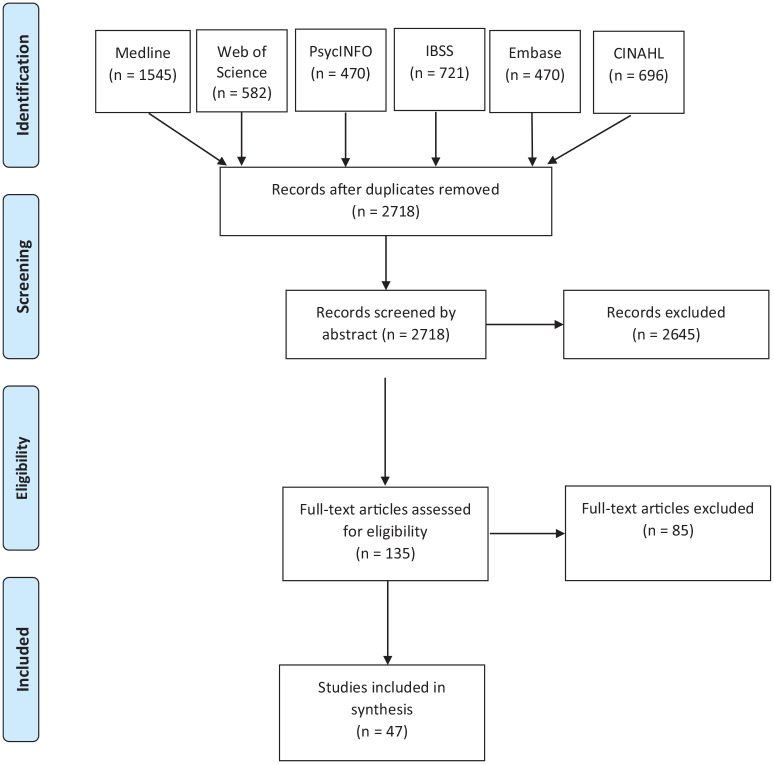
Flow diagram.

Our study maps Scandinavian efforts in promoting labour market inclusion of the chronically ill, with an aim to identify promising strategies and the need for further research. Reviews of a broad topic that include various study designs are categorised as a scoping study by Arksey and O’Malley [34]. This is in contrast to a systematic review that follows a specific research question based on specific study designs, also including a quality assessment of the studies under review. Scoping studies thus provide more of an overview with fewer details and less synthesis of results than a systematic review. Arksey and O’Malley [34] emphasise the rigorous and transparent mapping of a field of study as the main strength of this type of literature review. The authors present the lack of quality assessment and somewhat descriptive character as limitations. Scoping studies of this kind have similarities with what some refers to as a semisystematic review [35]. Scoping reviews are thus well suited to investigate areas where several research designs are applicable and have been used to explore various public health research questions, including national immunisation [36], precarious employment [37], end-of-life care [38], and migration health [39].Our strategy – including choice of databases, search strings, and inclusion criteria – was informed by the afore-mentioned previous reviews of similar areas. Figure 1 illustrates our search and screening strategy. The research note of Haafkens et al. [40] outlining useful principles for systematic reviews on chronic disease and work participation was also useful, as our search methodology aims at the same rigor and transparency as more systematic reviews. We performed searches in six databases dedicated to medical and social science literature: Medline, Web of Science, PsycINFO, International Bibliography of the Social Sciences (IBSS), Embase, and Cumulative Index to Nursing and Allied Health (CINAHL). We used the databases’ dedicated search engines, and the search results were handled in the reference management software Zotero. Search strings and inclusion criteria were adjusted after several initial exploratory searches. Inclusion and exclusion criteria are displayed in Supplemental Table A.1 and the search string is provided in Supplemental Table A.2, both tables are available in the Supplemental Appendix.

We included only empirical, peer-reviewed research articles investigating work inclusion of the chronically ill in the Scandinavian countries Sweden, Denmark and Norway. Grey literature and reports were excluded, as were review studies, commentaries and editorials, and study protocols. The final searches were performed on 10 February 2020, and included articles published after 1 January 2015. To access a broad knowledge base, we included various study designs: quantitative and qualitative, longitudinal and cross-sectional, trials and observational studies.

Work participation and chronic disease were the two vital components of our research question. The former concept was operationalised in our searches through various terms, such as ‘work capacity’, ‘work disability’, ‘vocational rehabilitation’, ‘occupational health’, ‘sick leave’, ‘absenteeism’, ‘return to work’, ‘retirement’, ‘employment status’ and ‘work status’, which also reflected the various potential outcomes of the studies we were interested in. The latter concept, chronic or non-communicable diseases, have had various definitions and operationalisations in previous literature reviews. Some reviews have limited themselves to specific diagnoses (cf. Nazarov et al. [14]), while others have shown a more functional approach by including any illness that may cause long-term work limitations and sickness absence (cf. Clayton et al. [22]). As we limited our institutional context to the Scandinavian countries and still wanted to access a broad array of studies, we therefore aimed at including all physical and mental conditions that could be regarded as chronic. Pilot searches revealed that the chronic nature of the studied conditions was not always explicitly stated. To include the word ‘chronic’ in the search string would therefore risk excluding relevant articles in which the chronic nature of the studied condition was implicit. We therefore chose to include chronic illness as an inclusion criterion to be assessed by the researchers, and not as a part of the search string. Further, work injuries were excluded if they did not have long-term consequences, that is, developed into a chronic condition.

We were primarily interested in interventions at the government (provided by the state and/or municipal services) or workplace level which aimed to improve work inclusion of people with chronic health conditions. Cross-country comparisons of the effect of macro-level measures on employment, such as countries’ net social spending or overall use of ALMPs, were not included. Initial searches also returned some studies that examined how employment outcomes were influenced by workplace characteristics such as social support from coworkers and perceptions of managerial styles, cf. reviews by Snippen et al. [27] and MacEachen et al. [19]. We therefore updated our criteria to exclude these studies, as their results did not imply external validity; we aimed at reviewing concrete interventions that could be implemented in different institutional settings. Our review does not cover direct employment by the government targeting people with chronic diseases and reduced work capacity.

## Results

The literature search returned a total of 4484 articles, reduced to 2718 after duplicates were removed. The 2718 articles were divided and screened by abstract only by the involved researchers. This initial screening resulted in 135 texts which were also divided and individually assessed by researchers through full-text reading. After the full-text assessment of the 135 texts, we ended up with a corpus of 47 articles. There were two main reasons for exclusion in the full-text screening: (a) articles did not address a specific intervention; and (b) they did not have work participation as an outcome (although often as a covariate). Such studies were challenging to exclude through the search strings, as we would then also exclude many relevant studies. The data we extracted from these articles is available in Supplemental Table A.3, and more detailed results are reported in Supplemental Table A.4. Our aim with this scoping review was to map the existing research on work inclusion of the chronically ill in Scandinavia. We therefore report results rigorously in Supplemental Table A.4, but do not attempt to synthesise the evidence in a meta-analysis or the like.

Of our 47 included studies, 28 were applied in a Norwegian context, 10 were from Sweden, seven from Denmark, and two [41, 42] were cross-country studies which included separate results from one or more Scandinavian countries.

Mental health issues were the most frequent chronic conditions, represented in 29 studies. These conditions included diagnosed depression, anxiety, acquired brain injury, behavioural disorders, psychotic disorders and schizophrenia, as well as broader categories such as common mental disorders, common mental complaints, severe stress, mood or anxiety disorders and severe mental illness. Musculoskeletal problems were also prevalent, included in 16 studies, mostly through general categories of ‘pains’, ‘problems’ and ‘disorders’. Seven studies focused on both mental health and musculoskeletal chronic conditions. One study investigated former cancer patients [43], and one study included specific results for cardiovascular conditions [44]. Finally, our corpus included 11 studies of catch-all categories of chronic disease, such as chronic pain, activity limitations and self-reported long-term illness.

Interventions were more challenging to classify and summarise, as several studies were dealing with interventions using and combining components from other programmes. We nevertheless attempted a tentative classification (see Table I). First, 10 articles studied mainly medically or clinically oriented interventions, such as cognitive-behavioural therapy [45]; physical exercise [46]; and clinical or inpatient programmes [47]. Second, in four articles, the interventions were directed at the employer, such as the possibility to adjust work demands [48]; measures of manager support [42]; or various workplace adaptions [49]. Third, 29 articles featured interventions that were neither medical/clinical nor directed at the workplace. This heterogenous group, for which we used the umbrella term ‘multimodal non-medical’, included studies of interventions such as individual placement and support [50]; occupational or vocational rehabilitation [51, 52]; multimodal forms of rehabilitation [53]; and education [54]. Finally, four articles studied interventions which did not fit with our classification, for example, reduced workload of older teachers [44] and cross-country comparisons of flexicurity policies [41].

**Table I.** Interventions.

Multimodal non-medical	Medical/clinical	Workplace	Other
Aas et al. [73] Aasdahl et al. [74] Bejerholm et al. [75] Brendbekken et al. [76] Brendbekken et al. [77] Evensen et al. [52] Falkum et al. [78] Farholm et al. [57] Gismervik et al. [79] Hamnes et al. [80] Hansen et al. [65] Haveraaen et al. [81] Hellman et al. [82] Hellström et al. [83] Høgelund and Falgaard Eplov [84] Johansen et al. [51] Jönsson et al. [85] Martin et al. [60] Pedersen et al. [61] Pietila-Holmner et al. [64] Porter et al. [58] Ree et al. [54] Rivano Fischer et al. [86] Rødevand et al. [50] Skagseth et al. [53] Skarpaas et al. [87] Thorsen et al. [43] Victor et al. [47] Werner et al. [63]	Braathen et al. [55] Hara et al. [88] Johanson et al. [59] Kaldo et al. [46] Knapstad et al. [56] Reme et al. [45] Skagseth et al. [89] Victor et al. [90] Wormgoor et al. [91] Øverland et al. [92]	Dellve et al. [48] Evans-Lacko and Knapp [42] Kuznetsova and Bento [49] Larsen et al. [93]	Backhans et al. [41] Bratberg et al. [44] Grahn et al. [94] Martin et al. [95]

As expected, the most common study outcome – utilised in 24 studies – was measures of work inclusion or RTW after absence or unemployment. Twelve studies also included sickness absence as an outcome, while 10 studies used various self-reports as outcomes, for instance self-perceived change in work ability [55]; work participation and functional status [56]; and need satisfaction, autonomous motivation, perceived competence, wellbeing and physical activity [57]. Our corpus also included some qualitative studies with less variable-oriented outcomes, such as critical factors in the RTW process [58]; enabling engagement in RTW [59]; and barriers and facilitators of a multidisciplinary, coordinated and tailored RTW intervention [60]. With regard to research design, 10 studies used qualitative or mixed methods, 17 were randomised controlled trials (RCTs), while 21 were other quantitative studies, such as cross-sectional and longitudinal cohort and survey studies.

Two studies reported a negative effect, that is, that the intervention increased the risk of not returning to work [61]. Approximately half – 25 – of the studies reported a clear positive impact for all or some of the participants in the intervention. Ten studies reported non-significant or very small effects, while 10 studies had a design which did not allow for a classification of positive or negative effects, for example, critical factors in the RTW process [58] or engagement in RTW [59]. Among the RCTs, the research design on top of the traditional evidence hierarchy, 10 of 17 studies reported a positive intervention effect, although with some heterogeneity across different outcomes. Out of these 10, seven were classified as multimodal non-medical interventions, while three were medical or clinical interventions. Among the other quantitative study designs, a majority of the multimodal non-medical interventions reported a positive effect, while the medical or clinical interventions showed some short-term, positive effects. This simple analysis suggests that interventions combining multiple elements in a non-clinical setting have some success in promoting work inclusion among the chronically ill. Table II displays the interventions stratified by country with the number of positive studies in parentheses. We can observe similar shares of positive effects across all countries and interventions.

**Table II.** Interventions by country (studies with positive results in parentheses).

	Norway	Sweden	Denmark	Other^a^	Total
Multimodal non-medical	18 (11)	6 (3)	5 (1)	0	29 (15)
Medical/clinical	8 (5)	2 (1)	0	0	10 (6)
Workplace	1 (0)	1 (1)	1 (0)	1 (1)	4 (2)
Other	1 (1)	1 (1)	1 (0)	1 (0)	4 (2)
Total	29 (17)	10 (6)	7 (1)	2 (1)	47 (25)

a Cross-country studies which included Scandinavian countries [41, 42].

## Discussion

Our review of published research on work inclusion of the chronically ill in the Scandinavian countries returned 47 relevant articles. The review demonstrates that mental health problems, followed by musculoskeletal conditions, were the most frequently studied chronic illnesses. In terms of interventions there are parallels between previous reviews and ours in addressing, for example, vocational rehabilitation, education, training and work placements and individual support measures (cf. Nazarov et al. [14] and Clayton et al. [31]). However, some reviews have returned more articles focusing on economic incentives (e.g. wage subsidies, benefits for welfare claimants, cf. Clayton and colleagues [22, 31]), while our review displays an overweight of interventions combining various elements such as education, physical exercise and more.

Mental health and musculoskeletal conditions are high on the political and research agenda in the Scandinavian countries. For instance, recent figures from the Norwegian Labour and Welfare Administration show how these two chronic conditions represent the two main diagnoses of people on sick leave in Norway, with a combined share of nearly 60% of lost workdays in 2020, 4th quintile [62]. Thus that numerous articles focus on mental health issues and musculoskeletal problems is not surprising, given the prominence of these chronic conditions in Scandinavian countries. The dominance of Norwegian studies (28 of 47 included studies) could reflect political concerns about sickness absence and work exclusion, where Norway stands out with relatively high levels especially for sickness, also in a Scandinavian context. In addition, Norway may have utilised ALMP and other policies to a lesser extent than Sweden and Denmark. As a consequence, funding in Norway may to a higher degree have been directed towards targeted measures and corresponding research on experiences with RTW and sickness absence studies.

What works? Our review indicates that relative to the other intervention types, there is a positive effect of multidisciplinary interventions. This was the case for RCTs as well as other study designs. In addition, Table II shows that these interventions and their ‘success rate’ are more or less equally distributed across the Scandinavian countries. Examples include Werner et al. [63], who describe an intervention consisting of educational meetings with physical therapists, guided physical exercise, individual consultation, weekly exercise and a follow-up consultation. Pietilä-Holmner et al. [64] describe a multimodal intervention based on a ‘bio-psycho-social approach’, including aspects such as physical exercise, relaxation, training in coping strategies and education in pain management. Hansen et al. [65] describe an intervention named GOBACK, consisting of medical consultations, ergonomic workplace adjustments and physical activity. Most of these interventions are directed at individual rehabilitation, with only a few directed at workplace adaptions. With a strong focus on interventions for individual employees, there is a risk of overlooking those with chronic illnesses without any previous attachment to the labour market. In addition, as noted by Clayton et al. [31], participants in these interventions may be more work-ready than the general population with chronic illness. Furthermore, interventions directed at the supply side of the labour market aim to improve individuals’ function at work or future ‘employability’. These interventions may risk overlooking the restraints or disincentives on the employer side. Several of the included studies describe locally implemented interventions aimed at a specific chronic illness and/or line of work. Although we have assessed them to have some external validity, these locally implemented interventions do not necessarily compensate for the excess costs associated with chronically ill employees. Governmental, all-encompassing programmes, potentially going beyond the traditional wage subsidies, may be better suited to incentivise employers to hire people with chronic diseases. On the other hand, these programmes are also acquired to consider work promotion. A common response to low employment rates among the chronically ill has been to create work incentives through cutbacks of benefits. Government programmes may therefore be more sensitive to the political and economic environment. Nevertheless, additional investigation of demand-side programmes and interventions may thus shed light on the more general strategies available for promoting work inclusion of the chronically ill.

What do our results tell us about existing research on work inclusion of the chronically ill in the Scandinavian countries? Whereas the attention on mental health and musculoskeletal conditions in our review comes as no surprise, we also noticed a significant lack of research on the effect of various governmental policies and programmes, including local health, work and welfare services. For a long time, policy documents and reforms have signalled the importance of a better coordination of different services better to serve employment and other needs of people with long-standing illnesses and conditions of multimorbidity [32, 66, 67]. Therefore, our review reveals that the vast sums invested in local health, welfare and work services have not received corresponding research attention in terms of their effect on labour market participation of the chronically ill. In addition, while policy goals stress the need for coordination between services and the use of cross-professional teamwork involving both health and work services professions, our review identifies a traditional division of labour between work and health interventions.

This may also reflect a limitation in our search and screening strategy. Von Simson (2019) has reviewed Norwegian research on public labour market interventions. The author found some peer-reviewed articles published post-2015 which evaluate governmental policies and programmes aimed at increasing labour market participation for people with health challenges. Examples include Hernæs [68], who found that activation requirements increase employment among young people; Markussen and Røed [69] who found that a comprehensive Norwegian activation programme, combining tailored rehabilitation, training and job practice, and a generous, stable, and non-means-tested benefit, has positive employment effects; and Salvanes et al. [70] who found that regular education as vocational rehabilitation has some short-term effect on employment for young people. Using Danish data, Holm et al. [71] found opposing effects of subsidised job training and education with regard to the obtainment and duration of employment. Using Swedish data, Fröhlich et al. [72] found no labour market effects of public rehabilitation programmes such as workplace training. These studies do not explicitly define their sample as chronically ill, nor do they display results for specific diagnoses; they tend to restrict their analyses to persons who are sick-listed or recognised by the welfare services with a disability or reduced capacity to work. In some cases, this status, in which a chronic disease very well may be the background, is a prerequisite for participation in these programmes. Our aim only to include articles that explicitly investigate work inclusion for the chronically ill may thus have excluded some evaluations of this type from our study corpus. We, however, argue that focusing our analysis on the chronically ill is expedient. This group often experiences labour market exclusion due to health challenges, without being ‘ill enough’ to qualify for the most comprehensive programmes, thus potentially falling between two stools.

What is the toolkit available to foster work inclusion for the chronically ill? Larger parts of the evidence describe small-scale supply-side interventions: medical or non-medical programmes directed at the individual employee. For many of these, results are positive, but we have questioned whether they represent long-term solutions in the process of increasing work participation among the chronically ill. Moreover, our findings indicate that research on work inclusion of people with long-standing illness is a field with a small evidence base in the Scandinavian context. This also reflects that much ‘testing’ is going on, wherein several interventions concentrate on ‘treatment’ of specific chronic condition groups. From this follows that other groups may lose out both in research and policy attention, for instance if their illnesses are long-standing, but less severe – or if they have little or no previous attachment to the labour market. These groups then ultimately risk that the issue of labour market inclusion is ‘privatised’ and that each must rely on their own individual resources.

## Supplemental Material

sj-docx-1-sjp-10.1177_14034948221096005 – Supplemental material for Promoting labour market inclusion of the chronically ill: a scoping review of Scandinavian countries’ efforts Click here for additional data file. Supplemental material, sj-docx-1-sjp-10.1177_14034948221096005 for Promoting labour market inclusion of the chronically ill: a scoping review of Scandinavian countries’ efforts by Håvard Thorsen Rydland, Henrik LitlerÉ Bentsen, Rune Ervik, Kjersti GrØnning, Kamrul Islam, Egil Kjerstad and Tord Skogedal LindÉn in Scandinavian Journal of Public Health
