# Supplementary material for: Promoting labour market inclusion of the chronically ill: a scoping review of Scandinavian countries’ efforts
Source: Scand J Public Health. 2022 May 10;51(8):1097–107. doi: 10.1177/14034948221096005 (PMC10642227; doi:10.1177/14034948221096005)
Supplement: sj-docx-1-sjp-10.1177_14034948221096005 – Supplemental material for Promoting labour market inclusion of the chronically ill: a scoping review of Scandinavian countries’ efforts [file sj-docx-1-sjp-10.1177_14034948221096005.docx]

# APPENDIX

**Table A.1: Inclusion and exclusion criteria**

| **Inclusion criteria** | **Exclusion criteria** |
| --- | --- |
| Studies published in 2015-2020 | Studies published before 2015 |
| Original, peer-reviewed research articles | White/grey literature  Reports |
| Studies set (not exclusively) in Norway, Sweden, Denmark | Studies not explicitly analyzing work inclusion in neither of the Scandinavian countries |
| English, Norwegian, Swedish, or Danish language | Other languages |
| Study design  Quantitative and qualitative  Longitudinal  Cross-sectional  Intervention/RCT  Treatment vs. no-treatment comparisons | Review studies  Commentaries and editorials |
| Outcome measure  Inclusion rate / probability of employment  Other work-related outcomes (commitment, motivation, satisfaction etc.)  Sickness absence  Wages / pensions | Health-related outcomes unrelated to work (e.g. mortality) |
| Physical and/or mental chronic condition  Self-reported and clinically diagnosed | Work injuries |
| Government policies/programs  Workplace interventions/programs (Medical/clinical/organizational etc. – can be difficult to separate)  Individual and environmental (employee and employer) focus | Macro-level measures like net social spending, economic downturns etc.  Articles not studying a specific policy/intervention/program |

**Table A.2: search string**

| **Thematic area** | **Search words** |
| --- | --- |
| Work inclusion | "work capacity" OR "work disability" OR "vocational rehabilitation" OR "occupational health" OR "sick leave" OR absenteeism OR "return to work" OR retirement OR "employment status" OR "work status" |
| Intervention | AND (service* or policy or policies or program*) |
| Country setting | AND (norway OR sweden OR denmark) |
| Time frame | AND 2015:2020 |

**Table A.3: Included articles: intervention, country, type of study, chronic condition, and type of outcome**

## Randomised controlled or clinical trials

| **Type of intervention(s)** | **Author(s)** | **Country(ies)** | **Type of study** | **Chronic condition(s)** | **Type of outcome(s)** |  |
| --- | --- | --- | --- | --- | --- | --- |
| Multimodal non-medical intervention:  Multidisciplinary, coordinated and tailored RTW intervention (detached from workplace) | Martin et al. (95) | Denmark | Quasi-experimental study (RCT) | Mental health | Recurrent sickness absence and unemployment two years after |  |
| Multimodal non-medical intervention:  In addition to the usual case management, intervention adds a multidisciplinary health assessment (MHA) performed by a job coach, a case manager, and a psychiatrist with occupational knowledge | Høgelund & Falgaard Eplov (84) | Denmark | Quasi-randomised controlled trial | Mental disorders | RTW |  |
| Multimodal non-medical intervention:  IPS modified for people with mood and anxiety disorders (IPS-MA) | Hellström et al. (83) | Denmark | Randomised clinical superiority trial | People with mood or anxiety disorder. | RTW and education |  |
| Multimodal non-medical intervention:  Multidisciplinary intervention (MI) Vs Brief intervention (BI) | Brendbekken et al. (76) | Norway | Randomised clinical trial | Chronic musculoskeletal pain | RTW – increased work participation |  |
| Multimodal non-medical intervention:  Multidisciplinary intervention (MI) vs. Brief intervention (BI) | Brendbekken et al. (77) | Norway | Randomised clinical trial | Chronic musculoskeletal pain, see p. 196 for details | RTW – increased work participation |  |
| Multimodal non-medical intervention:  3-month complex occupational intervention include medicine consultations, a work related evaluation and workplace intervention plan, workplace visit, and a physical activity program, over a single hospital consultation and an MRI | Hansen et al. (65) | Denmark | Randomised clinical trial | Low back pain | Cumulative self-reported sick leave during 6 months from baseline |  |
| Medical/clinical intervention:  Post-discharge boosted RTW telephone follow-up added to standard RTW follow-up | Hara et al. (88) | Norway | Randomised clinical trial | Chronic pain, chronic fatigue, or common mental disorders. | Entry to the ordinary work force analysed from time of discharge through 1 year |  |
| Multimodal non-medical intervention:  Inpatient multicomponent occupational rehabilitation program vs. outpatient rehabilitation | Aasdahl et al. (74) | Norway | Randomised clinical trial, but no control group | Musculoskeletal or mental disorders | Sickness absence and RTW |  |
| Multimodal non-medical intervention:  Multicomponent occupational rehabilitation, including physical activity | Skagseth et al. (53) | Norway | Randomised clinical trials | Sick listed due to musculoskeletal, psychological, or general/unspecified diagnosis | Physical activity (PA), sickness absence, RTW |  |
| Multimodal non-medical intervention:  Individual Placement and Support (IPS, experimental inter.) vs Traditional vocational rehabilitation (TVR, control intervention) | Bejerholm et al. (75) | Sweden | Randomised controlled trial | Severe mental illness | Vocational outcomes; primary employment rate, secondary working hours and weeks, job tenure, income, time to first employment (additional outcomes for community int.) |  |
| Multimodal non-medical intervention:  Inpatient multimodal occupational rehabilitation (I-MORE) | Gismervik et al. (79) | Norway | Randomised controlled trial | Musculoskeletal and common mental health disorders | The cumulative number of Sickness Absence (SA) days within 6 and 12 months follow-up. Time until sustainable RTW. |  |
| Multimodal non-medical intervention:  Workplace educational meetings | Ree et al. (54) | Norway | Randomised controlled trial | Neck and low back pain | Days of sick leave |  |
| Medical/clinical intervention:  Work-focused cognitive-behavioral therapy | Reme et al. (45) | Norway | Randomised controlled trial | Common mental disorders | Work participation at 12 months- follow-up |  |
| Medical/clinical intervention:  Workplace intervention added to an inpatient | Skagseth et al. (89) | Norway | Randomised controlled trial | Musculoskeletal, unspecified- or common mental health disorders | Sickness absence |  |
| Medical/clinical intervention:  Brief coping-focused psychotherapy (PsT) and short-term psychotherapy | Wormgoor et al. (91) | Norway | RCT | Common mental complaints | Sick leave as registered in Norwegian registries, comparison of two therapy schemes. |  |
| Medical/clinical intervention:  At Work and Coping – combination of components from cognitive behavioural therapy and principles from supported employment | Øverland et al. (92) | Norway | RCT | Common mental disorder | Income, work participation and benefit receival |  |
| Multimodal non-medical intervention:  Psycho-education program | Pedersen et al. (61) | Denmark | RCT: Individual data from four municipalities | Mental health or at risk of | Relative risk of full RTW |  |

## Cohort and other quantitative studies

| **Type of intervention(s)** | **Author(s)** | **Country(ies)** | **Type of study** | **Chronic condition(s)** | **Type of outcome(s)** |  |
| --- | --- | --- | --- | --- | --- | --- |
| Multimodal non-medical:  Education and exercise based self-management program. Nationwide: Better management of Patients with Osteoarthritis | Jönsson et al. (85) | Sweden | Quantitative, register data | Osteoarthritis (OA) | Several: Arthritis self-efficacy scale, pain frequency, use of OA medication, desire for surgery, fear-avoidance behaviour, physical activity, and sick leave reported at baseline, 3 and 12month |  |
| Multimodal non-medical:  Individually tailored RTW programmes with and without coordinator: occupational rehabilitation, treatment for medical or psychological issues, and follow-up and work clarification services | Skarpaas et al. (87) | Norway | Longitudinal cohort study | Full-time sick leave, with musculoskeletal and mental health disorders as most common reported diagnoses | Full and first RTW |  |
| Multimodal non-medical:  Interdisciplinary treatment program | Werner et al. (63) | Norway | Quantitative cohort study | Long-term lower back pains | Self-reported sickness absence and functional level through survey instrument |  |
| Multimodal non-medical:  Job Management Program (JUMP) – vocational rehabilitation program | Evensen et al. (52) | Norway | Intervention. Two experimental groups but no control group, participation is randomised. | Schizophrenia | Employment outcome, hours worked, work tenure |  |
| Multimodal non-medical:  JUMP | Falkum et al. (78) | Norway | Intervention. Two experimental groups and one group receiving treatment as usual. Participation is randomised. | Psychotic disorders, schizophrenia | Employment status – working/not working |  |
| Multimodal non-medical:  Multimodal rehabilitation in primary care | Pietila-Holmner et al. (64) | Sweden | Prospective longitudinal cohort study | Chronic pain | Sick leave |  |
| Multimodal non-medical:  Multimodal rehabilitation, “policy changes” | Rivano Fischer et al. (86) | Sweden | Longitudinal cohort study | Chronic pain | Sick-leave benefits |  |
| Multimodal non-medical:  Occupational rehabilitation | Aas et al. (73) | Norway | Longitudinal cohort study | Aquired brain injury (ABI) | Return-to-work (RTW) |  |
| Multimodal non-medical:  Occupational rehabilitation | Johansen et al. (51) | Norway | A non-randomised pre–post measures controlled design | Mental and behavioural disorders or diseases of the musculoskeletal system and connective tissue. | Changes in cognitive and emotional functioning (primary outcomes) and work and health measures (secondary outcomes) |  |
| Multimodal non-medical:  Rapid RTW program: patient education, group discussion, physical activity | Thorsen et al. (43) | Norway | Quantitative, survey based | Female cancer | Baseline characteristics of patients with unimproved work status |  |
| Multimodal non-medical:  Rapid-RTW program consists with multidisciplinary health personnel provide outpatient hospital service | Haveraaen et al. (81) | Norway | Cross-sectional cohort study | ABI | The development in patient and service characteristics the first 6 years of a RTW service for persons with ABI |  |
| Multimodal non-medical:  The treatment offered is individual, time-limited psychotherapy and/or psycho-educative courses for various problems such as depression, social phobia, panic disorder, stress and insomnia. | Victor et al. (47) | Norway | Prospective cohort study | Common mental disorder (CMD) | RTW 6 months after treatment |  |
| Multimodal non-medical:  Vocational rehabilitation program that partly draws on self-determination theory perspectives | Farholm et al. (57) | Norway | Quantitative. Longitudinal study (four time points over 15 months) | Musculoskeletal disorders (neck and back problems) and mental disorders | Need satisfaction, autonomous motivation, perceived competence, well-being, physical activity and RTW |  |
| Medical/clinical intervention:  Prompt Mental Health Care (Norwegian adaptation of English Improving access to psychological therapies IAPT) | Knapstad et al. (56) | Norway | Quantitative, prospective cohort design | Anxiety and depression | Work participation and functional status |  |
| Medical/clinical intervention:  RTW program in clinic (individual psychotherapy and/or psycho -educative courses for various problems such as depression, social phobia, panic disorders, stress or sleep problems) | Victor et al. (90) | Norway | Quantitative, pre-post study, medical records, and questionnaire data | Common mental disorders | Work participation: 1) Working fully; 2) working partly; 3) Not working; 4) No change; 5) working less |  |
| Medical/clinical:  1) Internet based cognitive behavioral therapy. 2) Supported physical exercise | Kaldo et al. (46) | Sweden | Quantitative, randomised controlled trial | Depression | Patients’ self-rating of employment status and long -term sick leave. |  |
| Workplace intervention:  Case management of sickness benefits cases based on private-public partnership | Larsen, Aust, & Høgelund (93) | Denmark | Quantitative, register data, difference in difference analysis | Not a focus on chronic conditions, but on sickness cases (so include a mix of chronic and non-chronic conditions) | Sickness duration, sickness duration until self-support |  |
| Workplace intervention:  Manager support | Evans-Lacko and Knapp (42) | 15 countries, including Denmark | Quantitative. Cross-sectional survey data | Self-reported depression | Level of absenteeism (days not working due to depression) and presenteeism score |  |
| Workplace intervention: Adjustment latitudes at work and home; work pace and workplace | Dellve, Fallman and Ahlstrom (48) | Sweden | Quantitative longitudinal. Questionnaires 3 times at a 6-month interval and after 6 years | Female human service workers on long-term sick leave – not a focus on one chronic condition, but on sick leave >60 days. The majority of participants had musculoskeletal and/or mental health disorders, stress symptoms and/or neck pain | Increased work ability and RTW |  |
| Other intervention:  Age dependent job condition - Reduced workload of older teachers | Bratberg, Holmås & Monstad (44) | Norway | Quantitative difference-in-difference | This is a study of one occupation – teachers – and not people with chronic conditions. However, the study provides information on psychiatric diagnosis, musculoskeletal diagnosis, and cardiovascular diagnosis | Sickness absence and health care utilization |  |
| Other intervention:  Nature-based rehabilitation program | Grahn et al. (94) | Sweden | Prospective Quasi-experimental study | Long-term reactions to severe stress and/or depression | RTW |  |

## Qualitative and mixed methods

| **Type of intervention(s)** | **Author(s)** | **Country(ies)** | **Type of study** | **Chronic condition(s)** | **Type of outcome(s)** | |  |
| --- | --- | --- | --- | --- | --- | --- | --- |
| Workplace intervention:  Workplace Adaptation | Kuznetsova and Bento (49) | Norway | Qualitative and quantitative (Multi-method approach). Interviews employers (in two large companies) and Norwegian Disabled People Labour Force Survey (LFS) (2006-2015) | Persons with disabilities (PwD), long-term illness | Workplace Inclusion | |  |
| Medical/clinical intervention:  The Individual Enabling and Support model (supported employment program). Combining Cognitive Behavioral Therapy, Motivational Interviewing, time use diary | Johanson et al. (59) | Sweden | Multiple- case design. Qualitative interviews with 5 participants and 2 int with employment specialist | Affective disorders (e.g. depression, bipolar disorder) | Enabling engagement in RTW | |  |
| Multimodal non-medical intervention:  Multimodal pain rehabilitation | Hellman et al. (82) | Sweden | Exploratory qualitative study using qualitative content analysis | Non-specific back pain | Rehabilitation and RTW. | |  |
| Multimodal non-medical intervention:  Return‐to‐work group programmes (RTW-GPs) | Hamnes et al. (80) | Norway | Focus group interviews and one individual interview | Musculoskeletal disorders | Remain in work and prevent absenteeism | |  |
| Multimodal non-medical intervention:  Traditional vocational rehabilitation. Individual enabling support | Porter et al. (58) | Sweden | Qualitative, in-depth interviews, 16 respondents | Depression | Critical factors in the return-to-work process | |  |
| Medical/clinical intervention:  Inpatient occupational rehabilitation program | Braathen et al. (55) | Norway | Qualitative, in-depth interviews, 17 respondents | Typical diagnoses are depression, anxiety, fatigue, low back pain, fibromyalgia, muscular pain conditions. No strict focus on chronic conditions, but on health problems and sickness absence. The study probably includes a mix of chronic and non-chronic conditions | Self-perceived change in work ability |  | |
| Multimodal non-medical intervention:  IPS | Rødevand et al. (50) | Norway | Qualitative, semi-structured interviews (8 participants) | Various chronic pain conditions | Patients’ experiences with IPS | |  |
| Multimodal non-medical intervention:  RTW intervention at three different sites (municipalities) | Martin et al. (60) | Denmark | Quantitative and qualitative data | Mental health | Barriers and facilitators of a multidisciplinary, coordinated, and tailored RTW intervention (detached from workplace) | |  |
| Other intervention:  Government policy - Flexicurity | Backhans et al. (41) | 21, including Scandinavian countries | Quantitative and qualitative. Longitudinal EU-SILC survey, Fuzzy set QCA | Activity limitations = “due to health problems that have lasted more than six months” (p.2) (self-reported) | RTW rates | |  |

**Table A.4: Included articles, results**

## Randomised controlled or clinical trials

| **Author(s)** | **Intervention vs. control** | **N** |
| --- | --- | --- |
| Martin et al. (95) | Mean differences:  Sickness absence, year one = Intervention group 58 days more; p<0.01  Sickness absence, year two = Intervention group 36 days more; p=0.03)  Self-supported at the end of follow-up = Intervention group 17% less; p=0.02 | Intervention group = 88  Control group = 80 |
| Høgelund & Falgaard Eplov (84) | HR sick leave duration for intervention group = 1.05; 95% CI 0.74–1.43  HR return-to-work duration for intervention group = 0.94; 95% CI 0.65–1.35 | Intervention group = 83  Control group = 99 |
| Hellström et al. (83) | RTW/education at 24 months  OR =1.34; 95% CI 0.86–2.10, p = 0.20  RTW/education at 12 months  OR = 1.19; 95% CI 0.74–1.92, p = 0.48 | Intervention group = 162  Control group = 164 |
| Brendbekken et al. (76) | 3-month partly RTW RR = 1.86; 95% CI 1.10–3.14  12-month partly RTW RR = 1.60; 95% CI 0.74–3.46 | Intervention group = 141  Control group = 143 |
| Brendbekken et al. (77) | 3-month RTW OR = 2.69; 95% CI 1.06–6.85  12-month RTW OR = 1.13; 95% CI 0.67–1.91 | Intervention group = 141  Control group = 143 |
| Hansen et al. (65) | Cumulative self-reported sick leave 6 months from baseline, mean difference in days = 3.50; 95% CI –5.08–12.07; p = 0.422  Cumulative self-reported sick leave OR = 0.94; 95% CI 0.53–1.66; p = 0.822 | Intervention group = 153  Control group = 152 |
| Hara et al. (88) | RTW OR = 1.87; 95% CI 1.06–3.31; p=0.031 | Intervention group = 104  Control group = 108 |
| Aasdahl et al. (74) | HR for sustainable RTW = 0.74; 95% CI 0.48–1.32; p = 0.165 (in favor of  outpatient program) | Inpatient group = 92  Outpatient (control) group = 76 |
| Skagseth et al. (53) | Increasing PA OR = 4.1; 95% CI 1.1–15.7  Consistently high PA OR = 3.1, 95% CI 1.0–9.7 | Intervention (short) = 92  Control (short) = 76  Intervention (long) = 86  Control (long) = 80 |
| Bejerholm et al. (75) | Differences in competitive employment:  Rate = 36; 95% CI 18–54  Weeks = 7.1; 95% CI 1.6–13.3  Workings hours = 176; 95% CI 66–302  Job tenure (weeks) = 7.3; 95% CI 0.9–14.0 | Intervention (short) = 60  Control (short) = 60  Intervention (long) = 41  Control (long) = 46 |
| Gismervik et al. (79) | Difference in median sickness absence = 32; p = 0.034  HR = 1.4; 95% CI 0.85–2.44; p = 0.17 | Inpatient group = 86  Outpatient (control) group = 80 |
| Ree et al. (54) | 3-month sick leave difference = 4.9 days; p = 0.001  6-month sick leave difference = 4.4 days; p = 0.016 | Intervention group = 646  Control group = 211 |
| Reme et al. (45) | 12-month work participation difference = 6.9%; p = 0.015  18-month work participation difference = 7.8%; p=0.018 | Intervention group = 437  Control group = 365 |
| Skagseth et al. (89) | 12-month median sickness absence difference = 15 days; p = 0.084  Sustainable RTW HR = 0.74; 95% CI 0.48–1.16; p = 0.192 | Intervention group = 88  Control group = 87 |
| Wormgoor et al. (91) | 3-month work participation difference = 9%; p = 0.039  24-month work participation difference = 4%; p = 0.87 | Intervention group = 141  Control group = 143 |
| Øverland et al. (92) | 3-year work rate difference  26 of 36 months = 0.007; 95% CI -0,064–0.077  24 of 36 months = 0.035; 95% CI -0.41–0,111  22 of 36 months = 0.045; 95% CI -0.028–0.118 | Intervention group = 630  Control group = 563 |
| Pedersen et al. (61) | 3-month RTW RR = 0.68; 95% CI 0.47–0.98  6-month RTW RR = 0.97; 95% CI 0.78–1.21 | Intervention group = 215  Control group = 215 |

## Cohort and other quantitative studies

| **Author(s)** | **Results and statistics** | **N** |
| --- | --- | --- |
| Jönsson et al. (85) | Difference in sick leave at 12 months:  “Knee” group = 9%; p<0.001  “Hip” group = 7%; p=0.017 | Knee group = 49415  Hip group = 22716 |
| Skarpaas et al. (87) | First RTW unadjusted HR = 0.70; 95% CI 0.53---0.94; p<0.02  Adjusted HR = 0.75; 95% CI 0.51---1.10; p<0.14  Full RTW unadjusted HR = 0.83; 95% CI 0.62---1.13; p<0.24  Adjusted = 0.82; 95% CI 0.55---1.22; p<0.32 | 326 |
| Werner et al. (63) | Improvement in Roland Morris Disability Questionnaire score (0-24:  4 weeks = 3.0; 95% CI 2,7–3,4; p<0.001  12 weeks = 4.0; 95% CI 3,6–4,5; p<0.001 | 433 |
| Evensen et al. (52) | RTW improvement similar between intervention groups, no statistics reported. | CBT intervention = 84  CR intervention = 64 |
| Falkum et al. (78) | 57.3% higher work participation in intervention group (p < 0.001) | Intervention group = 148  Control group = 341 |
| Pietila-Holmner et al. (64) | 3.9% increase in participants with no sickness absence at 1-year follow-up (p = 0.272) | 234 |
| Rivano Fischer et al. (86) | Participants without sick leave increased by 29% two years after intervention (p<0.001) | 7297 |
| Aas et al. (73) | RTW HR:  One-week group program = 0.598; 95%  CI: 0.395–0.906; p = 0.015  Meetings with the social insurance office = 0.473; 95% CI: 0.309–0.723; p = 0.001  Home visits = 0.523; 95% CI: 0.314–0.871; p = 0.013 | 137 |
| Johansen et al. (51) | 1.0 higher improvement in work ability (range 0-10) in intervention group (p = 0.001) | Intervention group = 280  Control group = 70 |
| Thorsen et al. (43) | 36% (95% CI 27–45) had unimproved work status at follow-up | 106 |
| Haveraaen et al. (81) | Delayed RTW:  High psychological job demands HR = 0.654; 95% CI 0.513–0.832  Low decision control HR = 1.297; 95% CI: 1.010–1.666 | 366 |
| Victor et al. (47) | Self-assessed work ability increased by 0.74 points; p = 0.003 | 106 |
| Farholm et al. (57) | RTW proportion change:  From time 1-3 = 20.5; p<0.001  From time 1-4 = 27.2; p<0.001 | Time 1 = 90  Time 2 = 80  Time 3 = 68  Time 4 = 57 |
| Knapstad et al. (56) | Proportion in work  Baseline = 40.0; 95% CI 38.5–43.6  Final treatment = 51.2; 95% CI 47.7-54.7  12 months post-treatment = 63.6; 95% CI 59.4–67.6 | Baseline = 1466  Final treatment = 789  12 months post-treatment = 533 |
| Victor et al. (90) | 8 % fewer participants not working post-treatment; p<0.001 | 164 |
| Kaldo et al. (46) | Unemployed subgroup evaluated on employment:  PE RR 3 months = 0.44; 95% CI 0.23---0.87  ICBT RR 3 months = 0.37; 95% CI 0.16---0.84  PE RR 1 year = 0.97; 95% CI 0.69---1.57  ICBT RR 1 year = 1.23; 95% CI 0.72---2.13  Employed subgroup evaluated on long-term sick leave:  PE RR = 1.4; 95% CI 0.52---3.74  ICBT RR = 0.99; 95% CI 0.39---2.46 | Initially unemployed (118)  Employed (703) |
| Larsen, Aust, & Høgelund (93) | Sickness benefit duration HR = 1.02, 95% CI 0.97–1.07  Duration until self-support HR = 0.99, 95% CI 0.96–1.02 | Intervention group = 110291  Control group = 71812  (Sickness benefit spells) |
| Evans-Lacko and Knapp (42) | Presenteeism score:  Countries where managers actively offer help = 7.08; 95% CI 6.59---7.58  Absent days:  Countries where managers avoid talking about = 4.13; 95% CI 1.68---6.57 | 16018 |
| Dellve, Fallman and Ahlstrom (48) | Increased work ability PR for 7-9 opportunities to adjustment latitudes = 1.56; 95% CI 1.05---2.34  Increased working degree PR for 4-6 opportunities to adjustment latitudes = 1.39; 95% CI 1.05---1.84 | 238 |
| Bratberg, Holmås & Monstad (44) | Significant intervention effects for men:  Monthly sick days = −0.31; p=0.06  Sick notes per year = −0.092; p=0.10 | Treatment group = 2449 men and 4860 women  Control group = 2650 men and 5620 women |
| Grahn et al. (94) | Increase in RTW in 8-week program = 20.0%; p<0.001  12-week program = 34.9%; p<0.0001  24-week program = 43.8; p<0.01 | 106 |

## Qualitative and mixed methods

| **Author(s)** | **N** |
| --- | --- |
| Kuznetsova and Bento (49) | Qualitative study = 12  Quantitative study N not reported |
| Johanson et al. (59) | 5 |
| Hellman et al. (82) | 15 |
| Hamnes et al. (80) | 17 |
| Porter et al. (58) | 16 |
| Braathen et al. (55) | 17 |
| Rødevand et al. (50) | 8 |
| Martin et al. (60) | Qualitative study = 4 group interviews, 10 individual interviews  Quantitative study = 213 |
| Backhans et al. (41) | Countries = 5  Individuals = 19881 |
